# A novel variant of ER-alpha, ER-alpha36 mediates testosterone-stimulated ERK and Akt activation in endometrial cancer Hec1A cells

**DOI:** 10.1186/1477-7827-7-102

**Published:** 2009-09-24

**Authors:** Sheng-Li Lin, Li-Ying Yan, Xing-Wei Liang, Zhen-Bo Wang, Zhao-Yi Wang, Jie Qiao, Heide Schatten, Qing-Yuan Sun

**Affiliations:** 1 State Key Laboratory of Reproductive Biology, Institute of Zoology, Chinese Academy of Sciences, Beijing, China; 2 Graduate School, Chinese Academy of Sciences, Beijing, China; 3 Center of Reproductive Medicine, Department of Obstetrics and Gynecology, Peking University Third Hospital, Beijing, China; 4 Department of Medical Microbiology and Immunology, Creighton University Medical School, Omaha, USA; 5 Department of Veterinary Pathobiology, University of Missouri, Columbia, MO 65211, USA

## Abstract

**Background:**

Endometrial cancer is one of the most common gynecologic malignancies and its incidence has recently increased. Experimental and epidemiological data support that testosterone plays an important role in the pathogenesis of endometrial cancer, but the underlying mechanism has not been fully understood. Recently, we identified and cloned a variant of estrogen receptor (ER) alpha, ER-alpha36. The aim of the present study was to investigate the role of ER-alpha36 in testosterone carcinogenesis.

**Methods:**

The cellular localization of ER-alpha36 was determined by immunofluorescence. Hec1A endometrial cancer cells (Hec1A/V) and Hec1A cells with siRNA knockdown of ER-alpha36 (Hec1A/RNAi) were treated with testosterone, ERK and Akt phosphorylation was assessed by Western blot analysis. Furthermore, the kinase inhibitors U0126 and LY294002 and the aromatase inhibitor letrozole were used to elucidate the pathway underlying testosterone-induced activities.

**Results:**

Immunofluorescence shows that ER-alpha36 was localized on the plasma membrane of the both ER-alpha- and androgen receptor-negative endometrial cancer Hec1A cells. Testosterone induced ERK and Akt phosphorylation, which could be abrogated by ER-alpha 36 shRNA knockdown or the kinase inhibitors, U0126 and LY294002, and the aromatase inhibitor letrozole.

**Conclusion:**

Testosterone induces ERK and Akt phosphorylation via the membrane-initiated signaling pathways mediated by ER-alpha36, suggesting a possible involvement of ER-alpha 36 in testosterone carcinogenesis.

## Background

Endometrial cancer represents one of the most common female pelvic malignancies and is the fourth most common type of cancer in North American and European women [1,2]. There are many risk factors for endometrial cancer, such as polycystic ovarian syndrome (PCOS), obesity, age at menopause, prolonged exposure to endogenous estrogens [3,4]. Recently, epidemiological studies have found that testosterone is associated with increasing endometrial cancer risk [5]. However, the molecular mechanism underlying testosterone carcinogenesis has not been established.

The Mitogen-activated protein kinase (MAPK) plays a key role in regulating cell differentiation and proliferation and provides protection against apoptosis [6]. MAPK is the pivotal component of intracellular phosphorylation cascades in both cytoplasm and the nucleus [7,8] and elevated MAPK activity has been detected in invasive breast carcinomas compared with the surrounding benign breast tissue [9]. Akt, also known as protein kinase B, is a well-characterized serine/threonine kinase that is activated by a variety of stimuli, including epidermal growth factor, insulin, heregulin, vascular endothelial growth factor or steroids, in a phosphoinositide-3-OH kinase (PI3K)-dependent manner [10-13]. Activated Akt promotes cell proliferation and survival by phosphorylating and modulating the activity of various transcription factors in the nucleus. Genetic and biochemical evidence suggest that aberrant activation of the PI3K/Akt pathway contributes to tumorigenesis, which is associated with a worse outcome [14]. The up-regulation of PI3K/Akt cascades is also found in human endometrial cancer tissues [15].

Recently, we identified and cloned a novel variant of estrogen receptor α with a molecular weight of 36 kDa that is transcribed from previously unidentified promoter located in the first intron of the original estrogen receptor α (ER-α66) gene [16]. ER-α36 differs from ER-α66 by lacking both transcriptional activation domains (AF-1 and AF-2), but it retains the DNA-binding domain and partial ligand-binding domains. It possesses a unique 27 amino acid domain that replaces the last 138 amino acids encoded by exons 7 and 8 of the ER-α66 gene. In the present study, we studied the ER-α36 function in endometrial cancer Hec1A cells, and explored the contribution of the MAPK/ERK and PI3K/Akt pathways mediated by ER-α36 to testosterone carcinogenesis.

## Methods

### Materials and reagents

Anti-ERK1/2 antibody, anti-phospho-ERK1/2 antibody (Thr^202^/Tyr^204^), anti-Akt antibody, anti-androgen receptor antibody, anti-estrogen receptor α antibody and anti-β-actin antibody were purchased from Santa Cruz Biotechnology (Santa Cruz, CA). Anti-phospho-Akt (Ser^473^) antibody was obtained from Cell Signaling Technology (Beverly, MA). Anti-aromatase antibody was purchased from Novus Biologicals (Novus Biologicals, Littleton, CO). ER-α36 specific antibody against the 20 unique amino acids at the C-terminal of ER-α36, was described before [16,17]. U0126 was purchased from Calbiochem (La Jolla, CA). LY294002, testosterone and estrogen were obtained from Sigma (St. Louis, MO). Letrozole was obtained from TRC (Toronto, Cananda).

### Cell culture and cell lines

Human ER-positive breast cancer MCF-7 cells and human prostate cancer LNCaP cells were obtained from American Type Culture Collection (Manassas, VA). MCF-7 cells were maintained at 37°C and 5% CO_2 _in DMEM (Gibcol-BRL, USA) with 10% fetal calf serum (Hyclone, UT). LNCaP cells were cultured in RPMI-1640 medium with 10% fetal calf serum and maintained at 37°C in a humidified atmosphere of 5% CO_2_. Human Hec1A endometrial cancer cells were provided by Dr. Li-Hui Wei (Peking University People's Hospital, Beijing). Hec1A cells were grown at 37°C with 5% CO_2 _in DMEM supplemented with 10% fetal calf serum. To establish stable cell line with ER-α36 expression knocked down by shRNA from Hec1A cells, we constructed an ER-α36 specific shRNA expression vector by cloning the DNA oligonucleotides5'-GATGCCAATAGGTACTGAATTGATATCCGTTCAGTACCTATTGGCAT-3' from the 3'UTR of ER-α36 cDNA into the pRNAT-U6.1/Neo expression vector from GenScript Corp. We established stable Hec1A cell lines transfected with an ER-α36 shRNA expression vector (Hec1A/RNAi) and the empty expression vector (Hec1A/V). Briefly, the ER-α36 shRNA expression vector pRNAT-U6.1/Neo plasmid containing the shRNA against ER-α36 and the empty expression vector were transfected into Hec1A cells with Lipofectamine 2000 (Invitrogen, Carlsbad, CA) according to the manufacturer's instruction as described elsewhere [17]. Forty-eight hours after transfection, cells were re-plated and selected with 600 μg/ml of G418 for two weeks. The medium was changed every three days until colonies appeared. Clones were pooled and expanded for further analysis. Hec1A/RNAi cell line is a mixture of more then twenty clones. A cell line with pooled clones transfected with the empty expression vector was termed Hec1A/V and used as a control.

### Immunofluorescence and confocal microscopy

The cellular localization of ER-α36 was determined by indirect immunofluorescence. Hec1A cells cultured on sterile glass coverslips were fixed in 4% paraformaldehyde in PBS for 10 min. After being permeabilized with 0.4% Triton X-100 for 10 min at room temperature, cells were blocked in 4% BSA-supplemented PBS for 1 hour and incubated overnight at 4°C with anti-ER-α36-specific antibody against the 20 unique amino acids at the C-terminal of ER-α36. After three washes in PBS, the cells were labeled with FITC-conjugated secondary antibody. The DNA dye Hoechst 33258 was used for nuclear staining. Microscopic analyses were performed using a Confocal Laser-Scanning Microscope (Zeiss LSM 510 META, Germany).

### Western blotting analysis

Cells were grown in phenol-red-free DMEM (Gibcol-BRL, USA) with 2.5% dextran charcoal-stripped fetal calf serum (Biochrom AG, Germany) for 48-72 hours and then switched to medium without serum 12 h before stimulation by the agents indicated. The cells were collected in ice-cold PBS, and the cell extracts were prepared in RIPA buffer with proteinase inhibitor cocktail from Sigma (St. Louis, MO). The protein concentrations of the cell lysates were determined and boiled with gel-loading buffer for 5 min at 100°C. Immunoblotting was performed as descibed previously [18]. Briefly, the proteins were separated by 10% SDS-PAGE and then transferred to polyvinylidene fluoride membranes. Following transfer, the membrane were blocked in TBST (TBS containing 0.1% Tween 20) containing 5% skimmed milk for 2 h, followed by incubation overnight at 4°C with appropriate primary antibodies. After washing three times in TBST, 10 min each, the membranes were incubated for 1 h at 37°C with 1:2000 horseradish peroxidase-conjugated appropriate secondary antibodies. Finally, the membranes were processed and visualized using the enhanced chemiluminescence detection system (Amersham, Piscataway, NJ).

## Results

### ER-α36 is expressed on the plasma membrane in Hec1A cells

**Figure 1:**
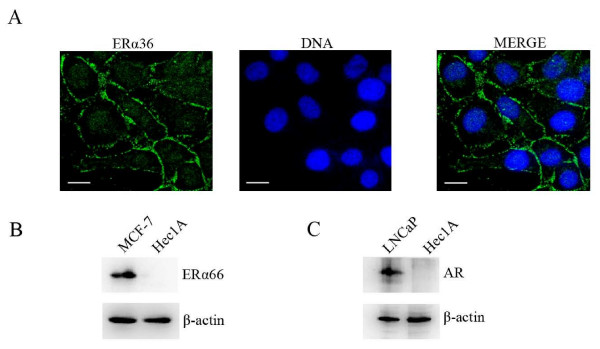
**Subcellular localization of ER-α36**. Hec1A cells were fixed and immunofluorescently stained with the anti-ER-α36 specific antibody against the 20 unique amino acids at the C-terminal of ER-α36 (green). The cells were counterstained with Hoechst 33258 (blue) to show the cell nuclei (A). Total protein was extracted from MCF-7, Hec1A and LNCaP cells and expression of ER-α66 (B) and androgen receptor (AR) (C) was analyzed by Western blot.

ER-α36 is a novel variant of ER-α66 generated by alternative promoter usage and alternative splicing [16]. To examine ER-α36 localization in Hec1A cells, immunofluorescencewas performed with anti-ER-α36 antibody raised against the 20 amino acids at the C-terminal of ER-α36 that are unique to ER-α36 [17]. Immunofluorescent staining revealed that ER-α36 is expressed on the plasma membrane of Hec1A cells (Figure 1A). It has been reported that endometrial cancer Hec1A cells are an ER-α66-negative cell line [19]. Consistent with this, Western blot analysis fails to detect the expression of ER-α66 (Figure 1B). Moreover, we found that Hec1A cells do not express androgen receptor (AR) (Figure 1C). Therefore, the endometrial cancer Hec1A cell line is an ER-α66-negative and AR-negative cell line.

### ER-α36 mediates testosterone-stimulated ERK activation

**Figure 2:**
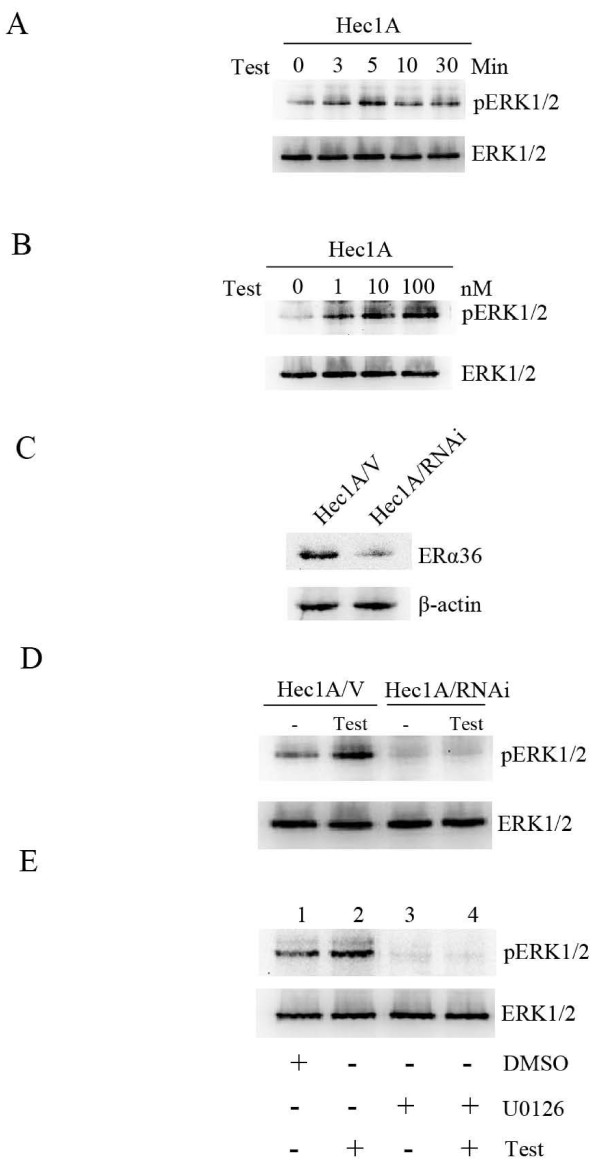
**ER-α36 mediates testosterone-stimulated ERK activation**. Time-course analysis of ERK1/2 phosphorylation in Hec1A cells treated with 10 nM testosterone (Test) for the indicated time points. The blot was stripped and re-probed with an anti-ERK1/2 antibody (A). Hec1A cells were treated 5 min with different concentrations of testosterone, and then lysates were immunoblotted with a phospho-specific antibody of ERK1/2. The same blot was stripped and probed with an anti-ERK1/2 antibody (B). (C), Western blot analysis of ER-α36 expression in Hec1A/V and Hec1A/RNAi cells. Western blot analysis of phospho-ERK1/2 in Hec1A/V and Hec1A/RNAi ER-α36 cells treated with 10 nM testosterone for 5 min. The same blot was stripped and probed with an anti-ERK1/2 antibody (D). Lysates were prepared from Hec1A cells treated with vehicle (DMSO) (Lanes 1), 10 nM testosterone (Lanes 2 and 4) or pre-treated with 10 μM U0126 (Lanes 3 and 4) for 30 min and immunoblotted with antibodies against phospho-ERK1/2 or total ERK1/2 (E).

MAPK/ERK signaling participates in the development and progression of many types of cancers including endometrial cancer [20]. To determine ER-α36 is involved non-genomic testosterone signaling in endometrial cancer cells, we first examined the phosphorylation levels of ERK, a serine-threonine kinase involved in cell proliferation [21]. As shown in Figure 2A, testosterone treatment induced phosphorylation of ERK1/2 in Hec1A cells. Re-probing the membrane with a total ERK1/2 antibody indicated that the total ERK1/2 content was not changed. We next examined the changes in ERK1/2 phosphorylation after treatment with different doses of testosterone. As shown in Figure 2B, testosterone induced a dose-dependent increase in ERK1/2 phosphorylation.

To test the involvement of ER-α36 in testosterone activity observed in Hec1A cells that lack ER-α66 and AR expression, we decided to knockdown ER-α36 expression with the siRNA approach. We established a stable cell line that expresses siRNA specifically against ER-α36 (Hec1A/RNAi) and found that ER-α36 expression was down-regulated in this cell line (Figure 2C). As shown in Figure 2D, testosterone failed to induce ERK1/2 phosphorylation in Hec1A/RNAi cells.

Extracellular regulated kinase kinase (MEK) acts upstream of ERK1/2 to phosphorylate and activate ERK1/2 [22]. The MEK specific inhibitor U0126 effectively inhibited the ERK1/2 activation stimulated by testosterone (Figure 2E). Our results indicated that the ER-α36-mediated Ras/MEK/ERK pathway is involved in testosterone signaling.

### ER-α36 mediates testosterone-stimulated Akt activation

**Figure 3:**
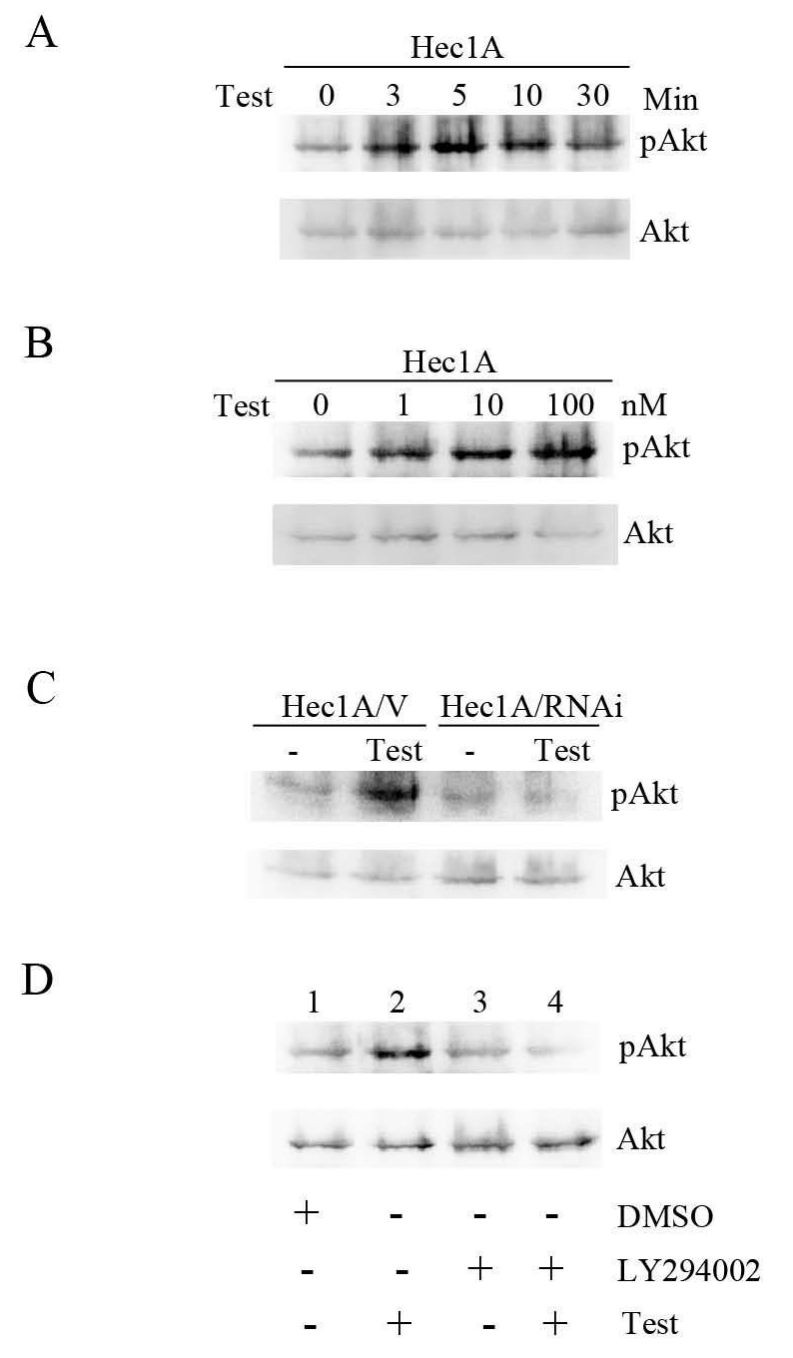
**ER-α36 mediates testosterone-stimulated Akt activation**. Western blot analysis of phospho-Akt in Hec1A cells treated with 10 nM testosterone (Test) for the indicated time points. The same blot was stripped and probed with an anti-total Akt antibody (A). Hec1A cells were treated 5 min with different concentrations of testosterone, and cell lysates were immunoblotted with a specific antibody against phospho-Akt. The same blot was stripped and probed with an anti-total Akt antibody (B). Western blot analyzed phospho-Akt in Hec1A/V and Hec1A/RNAi ER36 cells treated with 10 nM testosterone for 5 min. The same blot was stripped and probed with an anti-Akt antibody (C). Lysates were prepared from Hec1A cells treated with vehicle (DMSO) (Lanes 1), 10 nM testosterone (Lanes 2 and 4) or pre-treated with 50 μM PI3K inhibitor LY294002 (Lanes 3 and 4) for 1 h and immunoblotted with antibodies against phospho-Akt or total Akt (D).

The serine/threonine kinase Akt, or protein kinase B, plays an important role in cell proliferation and survival [23]. We then tested whether testosterone treatment induces Akt activation in Hec1A cells. As shown in Figure 3A, testosterone treatment induced the rapid phosphorylation of Akt. Furthermore, testosterone induced dose-dependent increase in Akt phosphorylation (Figure 3B). ER-α36 knockdown was able to abrogate testosterone-induced Akt phosphorylation, indicating the involvement of ER-α36 (Figure 3C). Pretreatment of Hec1A cells with the PI3K inhibitor LY294002 effectively inhibited Akt activation stimulated by testosterone (Figure 3D), indicating that testosterone regulates Akt phosphorylation through PI3K. Thus, our data indicated that ER-α36 is involved in testosterone-induced Akt activation.

### Letrozole inhibits ER-α36-mediated ERK and Akt phosphorylation

**Figure 4:**
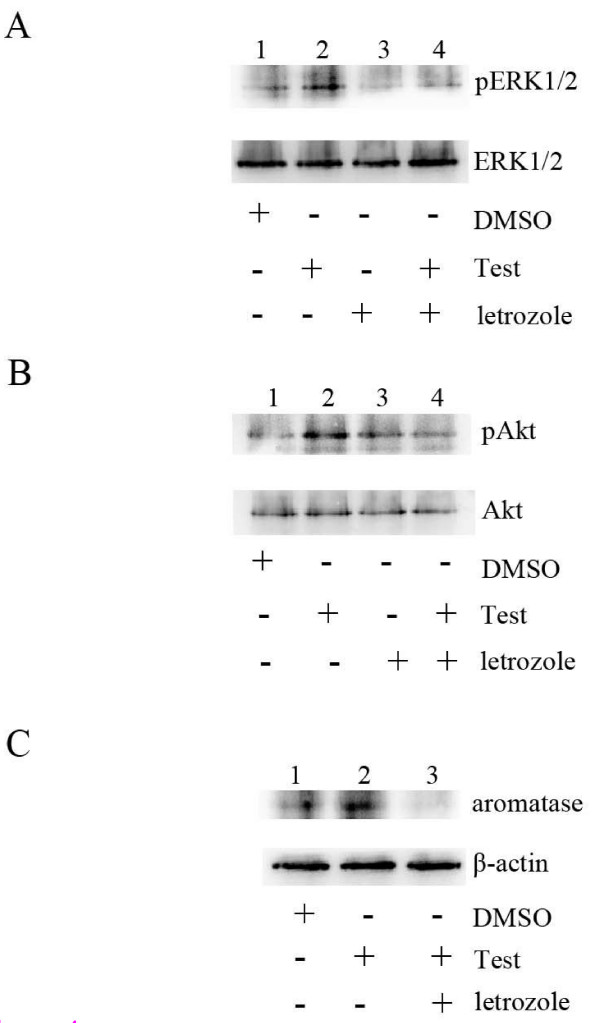
**Letrozole inhibits ER-α36-mediated ERK and Akt phosphorylation**. Hec1A cells were pre-treated with 10 nM aromatase inhibitor letrozole (Lanes 3 and 4) for 1 h and then the cells were treated with vehicle (DMSO) (Lanes 1) and 10 nM testosterone (Lanes 2 and 4) for 5 min. Phospho-ERK1/2 (A) or phospho-Akt (B) were examined with specific antibodies. The same blot was stripped and probed with anti-total ERK or Akt antibodies. Hec1A cells were treated with 10 nM testosterone (Lanes 2) or together with 10 nM aromatase inhibitor letrozole (Lanes 3) overnight, and then aromatase expression was detected by Western blot using specific antibody against aromatase (C).

Androgens are well-known to exert estrogenic effects via their aromatization to estrogens. Accumulating evidence suggest that estrogens are generated by in situ aromatization from cells of pathologically altered endometrium in postmenopausal women, which promotes malignant growth of these cells. Previous study also demonstrated that aromatase activity in the endometrium plays a vital role in the malignant transformation of endometrial cells by converting androgen into mitogenic estrogen in the endometrial tissue [24]. To determine the role of aromatase in non-genomic signaling pathway mediated by testosterone, we examined testosterone stimulated ERK and Akt phosphorylation in Hec1A cells pre-treated by letrozole, an aromatase inhibitor. As expected, letrozole abrogated the phosphorylation of ERK and Akt stimulated by testosterone (Figure 4A and 4B). In addition, we also found that letrozole treatment reduced expression levels of aromatase in Hec1A cells (Figure 4C). These data strongly suggest that aromatase is involved in testosterone activities in cells express ER-α36.

## Discussion

Estrogen receptor is a member of the nuclear receptor superfamily and function as ligand-dependent transcription factor in the nucleus to mediate estrogen signaling. However, accumulating evidence demonstrate that there is a rapid (within seconds or minutes) estrogen signaling which cannot be explained by genomic signaling pathway that usually takes hours to function [25,26]. Recently, we found that ER-α36 was expressed in ER-positive and ER-negative breast cancer cells [27], suggesting that ER-α36 expression is regulated differently from ER-α66. In the present study, we found that ER-α36 is expressed mainly on the plasma membrane in ER-α66-negative endometrial cancer Hec1A cells and ER-α36 mediates membrane-initiated MAPK/ERK and PI3K/Akt pathways induced by testosterone.

It has been reported that endometrial cancer risk is increased in both pre- and postmenopausal women with elevated plasma levels of testosterone [5,28]. Early in the neoplastic process, abnormal endometrial cells can locally produce estrogens from the plasma pool of androgen, and thus gain a growth advantage independent of circulating estrogens [29,30]. The local concentration of estrogens in endometrial cancer was reported to be higher than that in the blood and the endometrium of cancer-free women [31]. Indeed, previous studies have shown that aromatase activity is increased in endometrial cancer cells, but not normal endometrial cells [32]. Moreover, elevated circulating androgen has also been associated with hyperplasia of the endometrium, which generally precedes and accompanies the occurrence of type I endometrial carcinomas [33]. Aromatase is a key enzyme in the synthesis of estrogen that is responsible for binding of testosterone and catalyzes the series of reactions eventually resulting in estrogen production [34]. Previous reports demonstrated that aromatase is present in endometrial cancer tissue, suggesting that aromatase plays a role in converting testosterone into mitogenic estrogens in endometrial tissue [24,35]. Recently, a significant correlation has been found between aromatase immunoreactivity and poor prognosis in patients with endometrial carcinoma [36]. This positive linkage indicates that local aromatase contributes to tumor progression through the *in situ *formation of estrogens. Here, we show that testosterone stimulates the activation of both ERK1/2 and the Akt signaling pathways in endometrial cancer Hec1A cells that lack expression of ER-α66 and AR. Therefore, it is possible that the estrogen produced localy from testosterone in endometrial cells could bind ER-α36 and then activate MAPK/ERK and PI3K/Akt pathways.

PCOS is one of the most common endocrinopathies in humans, which affects about 10% of women of reproductive age [37]. PCOS is characterized by the production of endogenous progesterone and absence of ovulations and an increased secretion of ovarian androgen [38]. The association between PCOS and endometrial carcinoma has been reported for many years. The risk of development from PCOS to endometrial cancer was examined in 1270 women with chronic anovulation. This study identified the excess risk of endometrial cancer to be 3.1 [95% CI, 1.1-7.3] [33,39]. PCOS is a key risk factor especially for endometrial cancer among young, premenopausal women [40]. It is possible that increased rate by which androgen is converted to estrogen via aromatization, which then stimulates both the MAPK/ERK and the PI3K/Akt signaling pathways through ER-α36. The activation of ERK and Akt is involved the development of endometrial cancer [15,41].

Epidemiological, experimental and clinical result have shown that estrogen plays a key role in the development and progression of endometrial cancer [1]. Aromatase inhibitor inhibits local estrogen production in postmenopausal women and is used to treat postmenopausal women with breast cancer [42]. The large trials demonstrated that aromatase inhibitor contributed to improved disease-free survival and good tolerability in breast cancer patients [43]. Recently, aromatase inhibitor has been shown to reduce proliferation and increase apoptosis in endometrial cancer *in vitro *[44,45]. Letrozole is a competitive nonsteroidal aromatase inhibitor that suppresses over 85% of circulating levels of estrogen and over 98% of aromatization in postmenopausal patients with breast cancer [46]. In our study, we found that letrozole abrogated testosterone-induced ERK and Akt phosphorylation, suggesting that aromatase might be involved in testosterone carcinogenesis.

## Conclusion

In summary, we have shown that a novel variant of ER-α66, ER-α36 is localized on the plasma membrane of endometrial cancer Hec1A cells. We demonstrated that testosterone induces ERK and Akt phosphorylation via ER-α36-mediated membrane-initiated pathways. The present study thus shed new light on understanding testosterone-stimulated endometrial carcinogenesis. Further research of ER-α36 functions may provide novel information for designing new drugs for the treatment of endometrial cancer.

## Competing interests

The authors declare that they have no competing interests.

## Authors' contributions

SLL conducted the laboratory work and drafted the paper. LYY, XWL, ZBW, and JQ gave advice and reviewed the draft paper. ZYW provided the ER-α36 antibody, ER-α36 shRNA expression plasmid and control plasmid. HS and ZYW were also involved in manuscript preparation. QYS designed the study and amended the paper. All authors read and approved the final manuscript.
